# Prognostic Value of Neutrophil-to-Eosinophil Ratio (NER) in Cancer: A Systematic Review and Meta-Analysis

**DOI:** 10.3390/cancers16213689

**Published:** 2024-10-31

**Authors:** Taha Koray Sahin, Ruveyda Ayasun, Alessandro Rizzo, Deniz Can Guven

**Affiliations:** 1 Department of Medical Oncology, Hacettepe University, Ankara 06100, Turkey; 2 Department of Medicine, Icahn School of Medicine at Mount Sinai, New York, NY 10029, USA; ayasunr@gmail.com; 3 IRCCS Istituto Tumori “Giovanni Paolo II”, 70124 Bari, Italy; rizzo.alessandro179@gmail.com; 4 Medical Oncology Clinic, Elazig City Hospital, Health Sciences University, Elazig 23280, Turkey; denizcguven@hotmail.com

**Keywords:** biomarker, neutrophil-to-eosinophil ratio, meta-analysis, prognosis, survival

## Abstract

Background: The identification of reliable prognostic biomarkers is crucial for optimizing cancer treatment strategies, especially in the era of personalized medicine. This systematic review and meta-analysis evaluate the prognostic significance of the neutrophil-to-eosinophil ratio (NER) in various cancer types, with a focus on its association with overall survival (OS) and progression-free survival (PFS). Methods: We conducted a systematic literature search across PubMed, Scopus, and Web of Science databases for studies published up to 28 July 2024. We performed the meta-analyses with the generic inverse variance method with a random effects model and reported hazard ratios (HR) with 95% confidence intervals (CI). Results: The comprehensive literature search identified 10 studies comprising 2351 patients. Pooled analyses demonstrated that elevated pretreatment NER levels were significantly correlated with poorer OS (HR: 1.74, 95% CI: 1.28–2.36, *p* < 0.001) and PFS (HR: 1.53, 95% CI: 1.21–1.95, *p* < 0.001). Subgroup analyses confirmed a consistent adverse association between high NER and OS across various tumor types and geographic locations, although results from studies conducted in the Far East did not reach statistical significance. Conclusions: This meta-analysis demonstrates that elevated NER is associated with poorer OS and PFS in cancer patients, suggesting its potential utility as a non-invasive prognostic marker. Further validation in large, prospective studies is warranted to establish NER’s role in guiding personalized treatment strategies across diverse oncologic contexts.

## Simple Summary

The identification of robust prognostic biomarkers is paramount for refining cancer treatment strategies, particularly in the era of precision medicine. This systematic review and meta-analysis aim to assess the prognostic significance of the neutrophil-to-eosinophil ratio (NER) across various cancer types, with a specific focus on its correlation with OS and PFS. Pooled analyses demonstrated that elevated pretreatment NER levels were significantly associated with poorer OS and PFS. The study highlights NER as a promising and non-invasive biomarker that could help guide treatment decisions in cancer patients. However, further research is needed to confirm its potential across various cancer types and treatment settings.

## 1. Introduction

Cancer remains the second leading cause of death worldwide, despite substantial advancements in prevention, diagnosis, and treatment in the last few decades [1]. Although survival rates have improved for many cancer types, the overall burden of the disease continues to rise [2], and the total number of deaths from cancer is projected to grow from 10 million in 2022 to 16.3 million by 2040 [3,4] driven by an aging population and evolving risk factors. Furthermore, with improved overall survival, the number of cancer survivors is expected to increase significantly [5]. These survivors live with a variety of sequela due to the disease itself and the side effects of treatments [6]. Therefore, there is a growing interest to identify reliable biomarkers to enhance risk stratification and facilitate more personalized treatment escalation in patients with a higher risk of recurrence and treatment de-escalation for patients with a favorable prognosis to reduce toxicity [7].

Given the link between inflammation and the pathogenesis and progression of cancer, the prognostic significance of peripheral blood-based inflammatory markers has garnered increasing attention due to their advantages of convenience, low cost, and the potential of dynamic follow-up [8,9]. Several peripheral blood-based inflammatory markers, including the neutrophil-to-lymphocyte ratio (NLR), platelet-to-lymphocyte ratio (PLR), and pan-immune-inflammation value (PIV) have been demonstrated to predict the prognosis of patients with cancer [10,11,12]. The PIV is a composite marker calculated by multiplying the counts of neutrophils, platelets, and monocytes, then dividing by the lymphocyte count. This marker provides a comprehensive assessment of a patient’s systemic inflammatory and immune state, serving as a promising biomarker as a predictor of cancer outcomes [12]. However, compared to other complete blood count parameters, the prognostic role of eosinophils has been less extensively investigated, even though eosinophils play a crucial role in producing growth factors, cytokines, and chemokines forming tumor microenvironment [13,14]. Prior studies have reported pretreatment eosinophil count as a biomarker for improved response to immune checkpoint inhibitors (ICIs) in renal cell cancer (RCC), melanoma, and non-small cell lung cancer (NSCLC) [15,16,17].

Neutrophils and eosinophils, both integral components of the immune system, play distinct yet interconnected roles in cancer progression [18,19]. The balance between these two cell types, as reflected by the neutrophil-to-eosinophil ratio (NER), has emerged as a potential indicator of prognosis in various cancers [20,21]. In a pivotal study, a lower baseline NER was associated with improved clinical outcomes, including overall survival (OS), progression-free survival (PFS), and objective response rate (ORR) in patients with mRCC receiving ipilimumab and nivolumab [22]. ICIs, such as nivolumab and ipilimumab, are a class of immunotherapeutic agents that boost the anti-cancer immune response by blocking inhibitory pathways that cancers exploit to evade immune surveillance [23]. Subsequently, several studies have evaluated the correlation between NER and cancer prognosis, mostly in patients with mRCC receiving ICIs [24,25], although the tumor types, treatment types, methodology, cut-offs, and reporting varied considerably. Therefore, our study aims to summarize the available evidence on the association between the NER and survival outcomes in patients with cancer.

## 2. Materials and Methods

### 2.1. Search Strategies

This systematic review and meta-analysis were conducted in accordance with the Preferred Reporting Items for Systematic Reviews and Meta-Analyses (PRISMA) statement guidelines [26]. The protocol for this study was registered with PROSPERO ID CRD42024573779. The comprehensive literature search was conducted in the PubMed, Web of Science, and Scopus databases for relevant studies up to 28 July 2024. The keywords searched were “Neutrophil-to-eosinophil ratio” AND “carcinoma” OR “cancer” OR “chemotherapy” OR “immune checkpoint inhibitors”. Two investigators (TKS and RA) independently performed the search process and study selection, and any discrepancies regarding the eligibility of full-text articles were resolved by a senior author (DCG).

### 2.2. Eligibility Criteria

The inclusion criteria were as follows: (1) prospective or retrospective studies investigating the association between NER and PFS or OS; (2) categorization of NER into low and high groups; and (3) availability of hazard ratios (HR) and corresponding 95% confidence intervals comparing low NER and high NER groups. Exclusion criteria were as follows: (1) duplicate publications; (2) reviews, case reports, case series, guidelines, or editorials; (3) studies that evaluated NER as a continuous variable; (4) studies that did not report HR and CI for PFS or OS; and (5) publications in languages other than English.

### 2.3. Study Selection and Data Extraction

**Figure 1:**
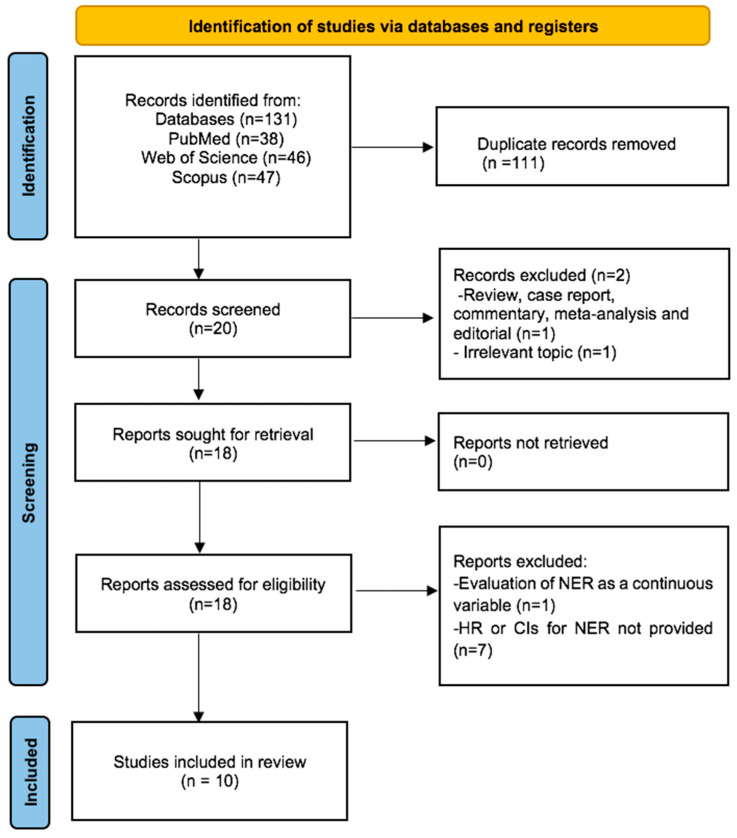
Flow diagram of included studies for this meta-analysis.

Our initial systematic search yielded a total of 131 records. After removing 111 duplicates, we screened the titles and abstracts of the remaining 20 records. We excluded 2 records due to an irrelevant topic (n = 1) and article type (review) (n = 1). The remaining 18 records underwent rigorous full-text review, and 8 studies were excluded for not meeting eligibility criteria (n = 1 for continuous NER, n = 7 for lack of HR and CIs for survival). Consequently, 10 studies were deemed eligible for quantitative synthesis. Figure 1 presents a detailed flowchart of the article selection process.

Two authors (TKS, RA) independently carried out data extraction in adherence to the meta-analysis of Observational Studies in Epidemiology (MOOSE) guidelines [27]. From each study, the extracted information included the lead author, country, year of publication, total sample size, patient distribution by low and high NER groups, cancer type, treatment regimen, and HR with 95% CIs for PFS or OS. The data from multivariable analysis was prioritized for extraction over univariable analysis whenever both estimates were available. Methodological quality and risk of bias were assessed using the Newcastle–Ottawa Scale (NOS).

### 2.4. Meta-Analysis

The primary outcome of interest was to assess the relationship between NER and OS in patients with cancer. The secondary outcomes were to evaluate the association between the PFS and the NER. Subgroup analyses were conducted taking into account geographic location (USA and Far East) and tumor type (genitourinary tumor vs. others).

Pooled analyses were conducted using the generic inverse variance approach under the random effects model, considering the heterogeneity across the studies. The effect sizes for OS and PFS were presented with HRs and 95% CIs. OS was defined as the time from treatment initiation to death or last follow-up, while PFS was defined as the time from treatment initiation to disease progression or death. Publication bias was evaluated using the Egger test by the funnel plot method. The heterogeneity was assessed within each subgroup using I^2^ statistics, with values over 50% considered indicative of significant heterogeneity. All meta-analyses were conducted using Review Manager (Revman, version 5.2 software; Nordic Cochrane Center, Copenhagen, Denmark). A *p*-value below 0.05 was considered statistically significant.

## 3. Results

### 3.1. Study Characteristics

This meta-analysis included a total of 10 studies [20,22,24,28,29,30,31,32,33,34] encompassing 2351 patients. One of the included studies was a post hoc analysis of a phase 3 JAVELIN 101 randomized controlled trial, while the remaining studies were retrospective analyses from real-world cohorts. The sample sizes spanned between 47 and 886. Geographically, five out of ten studies were carried out in the USA, followed by four studies conducted in the Far East. The most common cancer types were renal cell carcinoma (RCC) (n = 5) and head and neck cancers (n = 2). Eight studies had more than 80% of participants receiving ICIs either alone or in combination. Nine and seven studies reported data on the OS and PFS, respectively. The cut-offs were defined by ROC curve analyses in five studies, while median levels were used for patient dichotomization in four studies (Table 1). The NOS was used to assess methodological quality, with the majority of studies indicating a low risk of bias (Table 2).

### 3.2. Association Between NER Levels and Survival

**Figure 2:**
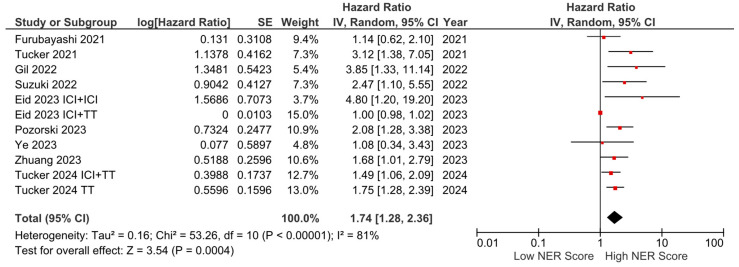
Forest plots of the association between NER and OS. Horizontal lines indicate 95% CIs. Diamond (♦) indicates the pooled effect size. The red squares represent the hazard ratios for each study.

Among the nine studies with OS data, six reported a negative effect of high NER levels on OS, while three studies reported no statistically significant association. In the pooled analysis of these studies, high baseline NER levels were associated with worse OS compared to low baseline NER levels (HR: 1.74, 95% CI: 1.28–2.36, *p* < 0.001) (Figure 2). The included studies had a high degree of heterogeneity (I^2^ = 81%). Sensitivity analyses were performed by sequentially excluding individual studies, yielding consistent results that support the robustness of our findings. The visualization of funnel plots pointed to no significant risk of bias (Figure S1).

**Figure 3:**
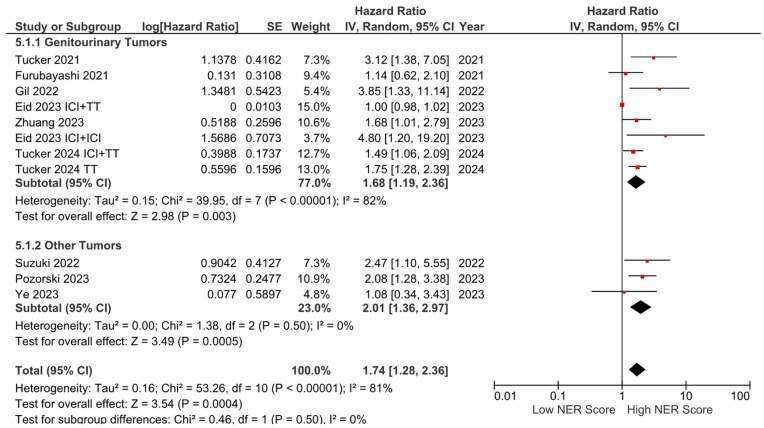
Subgroup analysis of overall survival based on cancer types.

**Figure 4:**
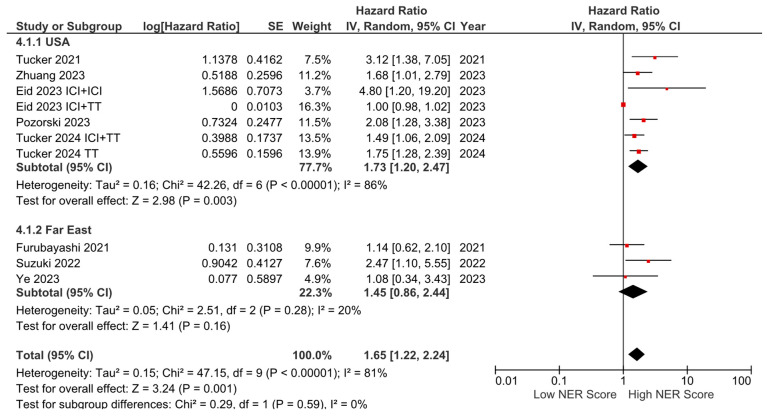
Subgroup analysis of overall survival based on country.

**Figure 5:**
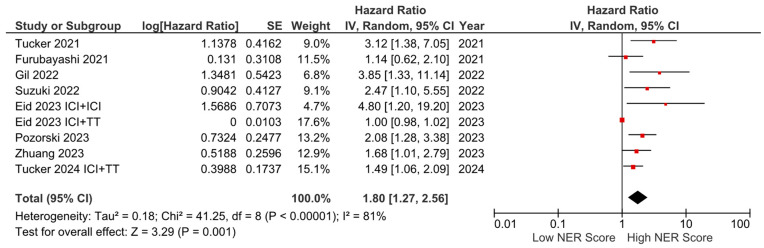
Subgroup analysis of overall survival in patients treated with immune checkpoint inhibitors.

Subgroup analyses for the tumor type (HR: 1.68, 95% CI: 1.19–2.36, *p* = 0.003 for genitourinary tumors; HR: 2.01, 95% CI: 1.36–2.97, *p* < 0.001 for other tumors) demonstrated a consistent negative association between high NER levels and OS (Figure 3). Subgroup analyses by study location revealed a similar trend across studies from the USA (HR: 1.73, 95% CI: 1.20–2.47, *p* = 0.003) and studies from the Far East (HR: 1.45, 95% CI: 0.86–2.44, *p* = 0.160) (*p*-value for subgroup differences = 0.590), although the analyses of studies from the Far East did not reach statistical significance (Figure 4). In patients receiving ICI-based regimens (eight studies), a significantly higher risk of death was observed in those with high NER levels compared to those with low NER levels (HR: 1.80, 95% CI: 1.27–2.56, *p* = 0.001) (Figure 5).

**Figure 6:**
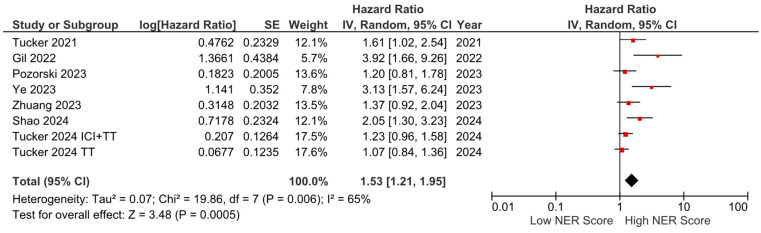
Forest plots of the association between NER score and PFS.

PFS data were available from seven studies and were included in the meta-analysis. The pooled analysis revealed that elevated NER levels were associated with worse PFS (HR: 1.53, 95% CI: 1.21–1.95, *p* < 0.001). The heterogeneity among studies was moderate, as indicated by an I^2^ statistic of 65% (Figure 6).

## 4. Discussion

In this meta-analysis, we observed a significantly higher risk of death and progression in patients with higher NER levels. The adverse effect of high NER in OS was consistent across tumor types and geographic locations. To the best of our knowledge, this study is the first meta-analysis investigating the association between NER values and survival in patients with cancer.

Inflammation plays a crucial role in tumorigenesis and development, with their relationship being both interdependent and synergistic [35]. Cytokine infiltration and high levels of inflammatory cells significantly contribute to carcinogenesis and tumor progression, potentially leading to severe malnutrition, impaired immune response, and tumor neovascularization over time [36]. Additionally, tumors often express high levels of certain growth factors and chemokines, which are released into the bloodstream, thereby modulating the levels of neutrophils, monocytes, lymphocytes, and eosinophils [37].

Numerous studies have demonstrated the crucial role of elevated neutrophils in tumor development and cancer prognosis [38]. Neutrophils can promote angiogenesis and remodeling of the extracellular matrix, thereby facilitating metastasis and tumor cell invasion [38]. However, eosinophils, a relatively scarce leukocyte subset, have historically been underappreciated [39]. The precise role of eosinophils within the tumor microenvironment (TME) remains to be fully elucidated, although emerging evidence suggests a potential anti-tumor function [13]. Eosinophils can migrate from the bloodstream into the TME through several integrin-mediated mechanisms [40]. Preclinical studies have demonstrated that activated eosinophils can enhance anti-tumor responses through various mechanisms, including recruitment of tumor-specific CD8+ T cells, normalization of tumor vasculature, and modulation of tumor-associated macrophages [41]. Furthermore, eosinophils have been implicated in anti-tumor activity through IL-33 and GM-CSF-IRF5 signaling pathways in specific cancer models [13,42].

A pioneering analysis from the tumor tissues of patients with colorectal cancer from the Iowa Women’s Health Study cohort demonstrated that tissue eosinophilia was inversely correlated with disease stage and was associated with a better OS and cancer-specific survival [43]. Furthermore, Yang et al. demonstrated a better OS and PFS in patients with in-tumor eosinophilia or patients with higher baseline or on-treatment peripheral blood eosinophilia, supporting that the peripheral blood could be a reflection of eosinophilia in tumor tissue [44]. Additionally, considering the widespread interactions between eosinophils and the adaptive immune system, the eosinophil could potentially synergize with ICIs to enhance immunotherapy efficacy [13], and there has been a strong interest in this topic in recent years. Indeed, elevated eosinophil counts have been associated with improved response to ICIs and prolonged OS in several cancer types [45,46]. Thus, eosinophilia has emerged as a promising biomarker in patients treated with ICIs. A recent study in NSCLC patients (n = 3143) showed that 770 patients (24.5%) had neutrophilia. Additionally, 108 patients (3.4%) had eosinophilia, while 75 patients (2.4%) exhibited both eosinophilia and neutrophilia [47]. Another study of HPV-associated cancers found that 31.9% of patients with anal squamous cell carcinoma and 42.6% of patients with oropharyngeal cancer had eosinophilia (defined as an eosinophil count <100 × 10^9^/L) [48]. These findings suggest significant deviations in neutrophil and eosinophil counts in patients with cancer, albeit with different cut-off values.

Given the role of neutrophils and eosinophils in cancer, a novel marker, NER, has been proposed as a significant prognostic indicator in patients with cancer. The findings of the pivotal study by Tucker et al. introduced the NER levels as a significant prognostic marker in patients with mRCC receiving nivolumab and ipilimumab [22]. Similarly, Zhuang et al. found that higher pretreatment NER levels were linked to worse OS in patients with mRCC treated with first-line ICI-based regimens [28]. Pozorski et al. also demonstrated that lower baseline NER was associated with improved OS and ORR in patients with advanced melanoma treated with ICIs, underscoring NER’s predictive value across various cancer types [20]. Beyond ICIs, the NER has demonstrated prognostic value in patients treated with surgery, chemoradiotherapy, or targeted therapy. Recently, Shao et al. demonstrated that NER has good predictive value for postoperative recurrence in patients with HCC [29]. Moreover, Ye et al. identified NER as an independent prognostic factor of distant metastasis-free survival in patients with advanced nasopharyngeal carcinoma receiving chemoradiotherapy, highlighting NER’s broad applicability across diverse oncologic settings [30]. Although direct comparisons of NER with other prognostic markers have been limited, Suzuki et al. revealed that NER was more consistently associated with improved clinical outcomes, such as PFS and OS, compared to the absolute eosinophil count and the relative eosinophil count [31]. Additionally, multivariate analysis identified only low NER (<32) as an independent prognostic factor for improved OS (*p* = 0.027). Among melanoma patients treated with ICIs, a lower pretreatment NER was significantly associated with enhanced ORR and OS, while the association between pretreatment NLR and OS did not remain significant following adjustment for clinical covariates [20].

There are several limitations of our study inherent to the retrospective nature of the included studies and the limited sample size in most studies. Additionally, the variability in cut-off values and cut-off selection methods for high and low NER across studies complicates direct comparisons and may affect the generalizability of our findings. The heterogeneity in tumor types, treatment regimens, and study methodologies further contributes to this complexity. Lastly, most of the studies included only patients treated with ICIs, limiting generalizability to patients treated with other modalities. However, despite these limitations, this meta-analysis provides the first evaluation of NER serving as a promising, non-invasive marker for predicting cancer outcomes. Addressing these limitations will necessitate future research efforts that establish standardized NER cut-off values and validation across larger, multicenter cohorts encompassing diverse treatment modalities beyond ICIs. Additionally, prospective studies monitoring NER fluctuations over time could provide insight into its utility as a dynamic predictor of treatment response and disease progression. Mechanistic studies of the immunomodulatory effects of NER in the tumor microenvironment (TME) will further elucidate its biological significance and may reveal new therapeutic targets.

## 5. Conclusions

In this meta-analysis, we observed that higher pretreatment NER levels were an independent factor associated with poorer OS and PFS, consistently across various tumor types and geographic regions. As an easily available and cost-effective serum biomarker, NER may help guide more personalized treatment decisions. Specifically, its incorporation into clinical practice could aid in stratifying patients by risk, thereby facilitating more individualized treatment planning. However, rigorous validation in large-scale prospective studies is essential to establish the clinical utility of NER in influencing treatment decisions across diverse oncologic contexts and therapeutic modalities.

## Figures and Tables

**Table 1:** Baseline characteristics of included studies.

Study	Year	Country	Sample Size	Cancer Type	Study Design	Cut-off Selection Method	NER Cut-Off Value	Sample Size (NER Low–High)	Primary Treatment	Tumor Stage	Survival Outcomes	Median Follow-Up (Months)
Tucker et al. [22]	2021	USA	110	RCC	Retrospective	Median	26.4	55–55	Nivolumab + Ipilimumab	Advanced	OS, PFS, ORR	19.6
Furubayashi et al. [32]	2021	Japan	105	Urothelial Carcinoma	Retrospective	ROC curve	13.7	24–81	Pembrolizumab	Advanced	OS	8.4
Gil et al. [24]	2022	Portugal	49	RCC	Multicenter retrospective	ROC curve	48	29–20	Nivolumab	Advanced	PFS, OS, ORR	9
Suzuki et al. [31]	2022	Japan	47	HNSCC	Retrospective	ROC curve	32	24–23	Nivolumab	Advanced	PFS, OS, ORR	N/A
Pozorski et al. [20]	2023	USA	182	Melanoma	Retrospective	Median	30.67	90–92	Anti-PD-1 monotherapy or combination therapy	Advanced	OS, PFS, ORR	26.7
Zhuang et al. [28]	2023	USA	184	RCC	Retrospective	ROC curve	49.2	138–46	ICIs	Advanced	OS, PFS, CBR	25.4
Eid et al. [33]	2023	USA	156	RCC	Retrospective	Median	21 (ICI + ICI)16.4 (ICI + VEGF-TT)	30–30 (ICI + ICI)–48–48 (ICI + VEGF-TT)	ICI-based regimens	Advanced	OS	N/A
Ye et al. [30]	2023	China	70	Nasopharyngeal Carcinoma	Retrospective	ROC curve	29.6	33–37	Neoadjuvant Chemotherapy + CRT	Advanced	OS, DMFS	N/A
Shao et al. [29]	2024	China	562	HCC	Retrospective	X-tile 3.6.1 software analysis	102	494–68	Hepatectomy	Localized	PFS	61.0
Tucker et al. [34]	2024	USA	886	RCC	Post hoc analysis of JAVELIN 101	Median	28	191–192 (ICI + ICI)–195–201 (TT)	Avelumab + Axitinib or Sunitinib	Advanced	PFS, OS, ORR	28+

Abbreviations: CRT: chemoradiotherapy; ICIs: immune checkpoint inhibitors; HCC: hepatocellular carcinoma; HNSCC: head and neck squamous cell carcinoma; OS: overall survival; PFS: progression-free survival; RCC: renal cell carcinoma; TT: targeted therapy; VEGF: vascular endothelial growth factor.

**Table 2:** The Newcastle–Ottawa Scale for assessing the quality of studies in meta-analysis.

Author, Year	Selection	Comparability	Exposure/Outcome	NOS Score
Tucker, 2021 [22]	****	**	***	9
Furubayashi, 2021 [32]	***	**	**	7
Gil, 2022 [24]	***	**	**	7
Suzuki, 2022 [31]	***	**	***	8
Pozorski, 2023 [20]	****	**	**	8
Zhuang, 2023 [28]	***	**	***	8
Eid, 2023 [33]	No full-text available			
Ye, 2023 [30]	***	**	***	8
Shao, 2024 [29]	****	**	**	8
Tucker, 2024 [34]	****	**	***	9

Each asterisk is equivalent to one point. The maximum score is 9 (**** for selection, ** for comparability, *** for outcome). Score of 5 to 6 considered as moderate quality and 7 to 9 as high quality.

## Data Availability

The data that supports the findings of this study are available in the manuscript.

## Supplementary Materials

The following supporting information can be downloaded at: https://www.mdpi.com/article/10.3390/cancers16213689/s1, Figure S1: Funnel plots of publication bias assessment for the studies on overall survival (OS).
