# Supplementary material for: Prognostic Value of Neutrophil-to-Eosinophil Ratio (NER) in Cancer: A Systematic Review and Meta-Analysis
Source: Cancers (Basel). 2024 Oct 31;16(21):3689. doi: 10.3390/cancers16213689 (PMC11545344; doi:10.3390/cancers16213689)
Supplement: Supplementary file 1 [file cancers-16-03689-s001.zip › supplementary-figure S1.pdf]

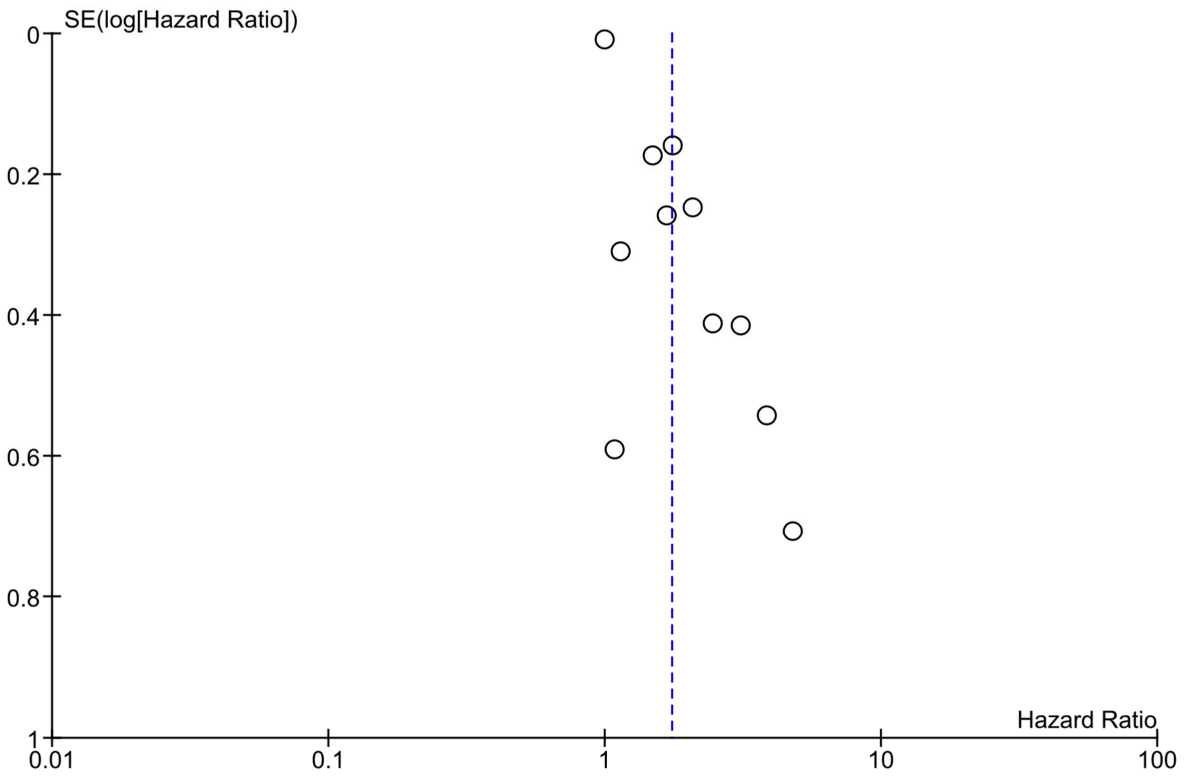

Supplementary Figure S1. Funnel plots of publication bias assessment for the studies on overall survival (OS). Supplementary Figure 1. Please add to the figure legend what each dot and the midline indicate for general readers.
